# Impact of NF-κB and reactive oxygen species on intracellular BAFF/APRIL expression in ANCA-associated vasculitis: focusing on the effect of resveratrol

**DOI:** 10.3389/fimmu.2025.1586158

**Published:** 2025-06-04

**Authors:** Yasuhiro Shimojima, Dai Kishida, Takanori Ichikawa, Yoshiki Sekijima

**Affiliations:** Department of Medicine (Neurology and Rheumatology), Shinshu University School of Medicine, Matsumoto, Japan

**Keywords:** ANCA-associated vasculitis, BAFF, APRIL, resveratrol, NF-κB, reactive oxygen species

## Abstract

Increased expression of B-cell activation factor of the tumor necrosis factor family (BAFF) and a proliferation-inducing ligand (APRIL) expression, which have been observed not only in the active phase of antineutrophil cytoplasmic antibody (ANCA)-associated vasculitis (AAV) but also in remission, may cause relapse by activating autoreactive B cells that produce ANCA. It is necessary to identify a therapeutic target related to the production of BAFF and APRIL in immune cells, particularly monocytes which play a crucial role in mediating the pathological processes of AAV. We previously demonstrated the efficacy of resveratrol (RVL) in restoring the function of regulatory T cells in AAV. This study examined the effects of RVL on the expression of BAFF and APRIL in monocytes as well as their related signaling factors in patients with AAV. This study used peripheral blood mononuclear cells (PBMCs) from 35 patients with AAV and 22 healthy controls. After incubating PBMCs with and without RVL or tempol, BAFF, APRIL, reactive oxygen species (ROS), and nuclear factor-κB (NF-κB) in CD14+ cells were analyzed using flow cytometry. Additionally, BAFF and APRIL in CD14+ cells were assessed in PBMCs treated with the NF-κB inhibitor, SN50. Significantly higher BAFF, APRIL, ROS, and NF-κB expression were observed in CD14+ cells in patients with AAV than in healthy controls. In CD14+ cells treated with RVL, patients with AAV exhibited significant increases in BAFF and NF-κB expression but significant decreases in APRIL and ROS expression. In patients with AAV, there was a positive correlation between NF-κB and BAFF in CD14+ cells, regardless of RVL treatment. Patients with AAV showed a significant decrease in APRIL expression without significant changes in BAFF expression in CD14+ cells treated with tempol; whereas there was a significant decrease in BAFF and APRIL expression in CD14+ cells treated with SN50. NF-κB may be a crucial signaling factor in BAFF production. RVL induces BAFF expression in monocytes by stimulating NF-κB in AAV, while its redox reaction reduces APRIL expression.

## 1 Introduction

Antineutrophil cytoplasmic antibody (ANCA)-associated vasculitis (AAV) is a systemic autoimmune disorder that is clinically categorized into three types: microscopic polyangiitis (MPA), granulomatosis with polyangiitis (GPA), and eosinophilic granulomatosis with polyangiitis, which can lead to serious or irreversible damages. AAV is pathologically characterized by a pauci-immune and necrotizing small- to medium-sized vasculitis (1, 2). Moreover, immunological cross-talks participates in the development of inflammatory vessel damage (2, 3). This process involves the participation of ANCA targeting myeloperoxidase (MPO) and proteinase 3 (PR3) via innate immune priming. Activation of neutrophils and the complement pathway leads to the promotion of neutrophil extracellular traps and an increased production of proinflammatory cytokines, chemokines, and reactive oxygen species (ROS). Multiple factors, such as polygenic genetic susceptibility, epigenetic impairment, and several environment factors, have also been suggested in the development of AAV. MPO- and PR3-ANCA have been found to be pivotal in the pathogenesis of AAV, suggesting that the activation signal of the B-cell lineage is also potentially involved in producing ANCA as an upstream factor in the pathological signaling pathway.

B-cell activation factor of the tumor necrosis factor family (BAFF) and a proliferation-inducing ligand (APRIL) are well-known as survival and activating factors for B-cells (4–6). Increased production of BAFF and APRIL has been identified in AAV (7–10) and is significantly correlated with circulating B cell expression and the expression of relevant receptors in patients with AAV (11). Additionally, significantly increased serum BAFF and APRIL levels have been still observed even in the remission phase of AAV (10–12), suggesting that persistent increases in BAFF and APRIL levels may cause relapse by activating B cells, leading to the production of ANCA. In real-world practice, some practical antagonists for BAFF, APRIL, and their binding receptors, such as belimumab, blisibimod, tabalumab, or atacicept (13–16), can be considered as the potential remedies. The efficacy of belimumab can be achieved under concomitant use of rituximab, although belimumab alone with conventional immunosuppressive agents does not reduce AAV relapse (17). This suggests that autoreactive B cells remain persistently activated in the environment with high production of BAFF and APRIL. Rituximab has enough clinical evidence supporting its effectiveness for both inducing remission and maintaining therapy in AAV (18–20), indicating that depleting autoreactive B cells, even those activated by persistent BAFF/APRIL expression, may be one of the most useful therapeutic strategies in AAV. However, administration of rituximab requires regular repetition to maintain remission, which leads to adverse effects, particularly frequent infections. It may be necessary to identify a therapeutic method that fundamentally inhibits the production of BAFF and APRIL by immunocompetent cells, including monocytes, to prevent the activation of B cells due to the involvement of BAFF and APRIL. To the best of our knowledge, the distinct intracellular signaling pathways that lead to the production of BAFF and APRIL remain to be elucidated.

We have previously demonstrated the efficacy of resveratrol (RVL), a phenolic compound with antioxidant, anti-inflammatory, and anti-immune aging properties, in restoring the regulatory function and recovery from effector plastic changes in regulatory T cells in AAV (21). Moreover, the suppression of increased intracellular ROS expression by RVL treatment was useful for repairing regulatory T cell function in patients with AAV. Herein, we assume that RVL may also be useful for regulating enhanced intracellular signaling of BAFF/APRL production, potentially through redox reactions. In this study, we assessed the influence of RVL on intracellular BAFF and APRIL expression in the circulating monocytes of patients with AAV. Additionally, the pivotal intracellular signaling factors for BAFF and APRIL production were identified through theexperiments with RVL treatment. Moreover, we focused on the expression of NF-κB in monocytes because it is a crucial intracellular factor involved in producing several cytokines, which can be modulated by ROS (22, 23).

## 2 Materials and methods

### 2.1 Patients and samples

A total of 35 patients (median age: 71 years; 22 women) without any immunosuppressive treatment, 21 with MPA and 14 with GPA, were enrolled in this study. The classification of MPA or GPA was determined according to the criteria of the Chapel Hill Consensus Conference (1), the consensus algorithm proposed by the European Medicines Agency (24), and/or the 2022 American College of Rheumatology/European Alliance of Associations for Rheumatology classification criteria (25, 26). The median (interquartile range, IQR) Birmingham Vasculitis Activity Score (BVAS) (27) was 15 (9–19). Relevant manifestations based on BVAS and laboratory findings, including the number of white blood cells, neutrophils, and lymphocytes; serum levels of C-reactive protein; estimated glomerular filtration rate (eGFR); and positivity for MPO-ANCA or PR3-ANCA, were also evaluated before initiating treatment (**Supplementary Table 1**). Peripheral blood samples were collected simultaneously from all the patients. Peripheral blood samples were also obtained from 22 healthy controls (HC), whose median age (64 years) and sex (12 women) were not significantly different from those of patients with AAV (*p* = 0.085 and *p* = 0.587, respectively). This study was approved by the local ethics committee of Shinshu University (approval number: 5787). All the participants provided written informed consent.

### 2.2 Isolation of blood samples and incubation

Peripheral blood mononuclear cells (PBMCs) were isolated from whole blood samples collected in ethylenediaminetetraacetic acid-coated tubes through gradient centrifugation using Ficoll-Hypaque PLUS (GE Healthcare, Pittsburgh, PA, USA). PBMCs were incubated on a 24-well plate (1 × 10^6^/well) with and without 100 μM RVL (Sigma-Aldrich, St. Louis, MO, USA) at 37°C for 24 h based on the method that we previously described (21). Alternatively, for reducing intracellular ROS, PBMCs were incubated on a 24-well plate (1 × 10^6^/well) with and without 50 μM tempol at 37°C for 24 h based on our previous experiment (28). The dosages of RVL and tempol were established through preliminary experiments in our previous studies as the most effective for reducing ROS without causing toxic adverse effects. In the experiment aimed at inhibiting intracellular nuclear factor-κB (NF-κB), 18 μM SN50 (Selleck Chemicals, Houston, TX, USA) was added simultaneously while incubating PBMCs with RVL, a dosage commonly used in related previous experiments (29, 30).

### 2.3 Cell treatment and flow cytometry

Incubated PBMCs with and without RVL, tempol, or SN50 were stimulated with 0.5 mg/mL lipopolysaccharide (LPS) (Sigma-Aldrich) and 2 μM monensin (BD Biosciences, San Diego, CA, USA) at 37°C for 4 h. Stimulated PBMCs were stained with FITC-conjugated anti-CD14 (BD Biosciences, San Diego, CA, USA). The stained PBMCs were permeabilized with Cytofix/Cytoperm (BD Biosciences) and then stained with PE-conjugated anti-BAFF (BioLegend, San Diego, CA, USA), APC-conjugated anti-APRIL (Miltenyi Biotec, Bergisch Gladbach, Germany), and PE/Cy7-conjugated anti-NF-κB (BD Biosciences) (**Supplementary Table 2**). Alternatively, for measuring cellular ROS expression, incubated PBMCs with and without RVL or tempol were stimulated with 200 μM tert-butyl hydroperoxide at 37°C for 60 min. Treated PBMCs were stained with CellROX Deep Red Reagent (Invitrogen, Carlsbad, CA, USA) and FITC-conjugated anti-CD14. The proportion and median fluorescence intensity (MFI) of targeted markers in the gated CD14+ population (**Supplementary Figure 1**) were evaluated. Stained cells were acquired on a FACSCanto II flow cytometer (BD Bioscience), and the data were analyzed using FlowJo software version 10.5.3 (Tree Star Inc., Ashland, OR, USA).

### 2.4 Statistical analysis

All data are presented as medians and interquartile range (IQR). Statistical significance was set at *p* < 0.05. The Mann–Whitney U test was used to compare two independent groups. The Kruskal–Wallis test was performed to compare three independent groups, and the Steel–Dwass test was subsequently used for multiple comparisons. Consecutive data, with and without treatment, were compared using the Wilcoxon signed-rank test and two-way analysis of variance. Regression analyses were used to evaluate the associations of BAFF and APRIL in CD14+ cells with BVAS or relevant manifestations. We estimated a partial regression coefficient (coefficient) or odds ratio (OR), respectively, together with a 95% confidence interval (CI). The Spearman’s rank correlation coefficient test was performed to evaluate the association of BAFF and APRIL expression with NF-κB expression in CD14+ cells. The cutoff thresholds for the proportion of BAFF or APRIL in CD14+ cells were calculated using the receiver operating characteristic (ROC) curve. Statistical analyses were performed using JMP software version 14.3.0 (SAS Institute Inc., Cary, NC, USA) and BellCurve for Excel (SSRI, Tokyo, Japan).

## 3 Results

### 3.1 BAFF and APRIL expression in CD14+ cells and their impacts on clinical findings in AAV

**Figure 1:**
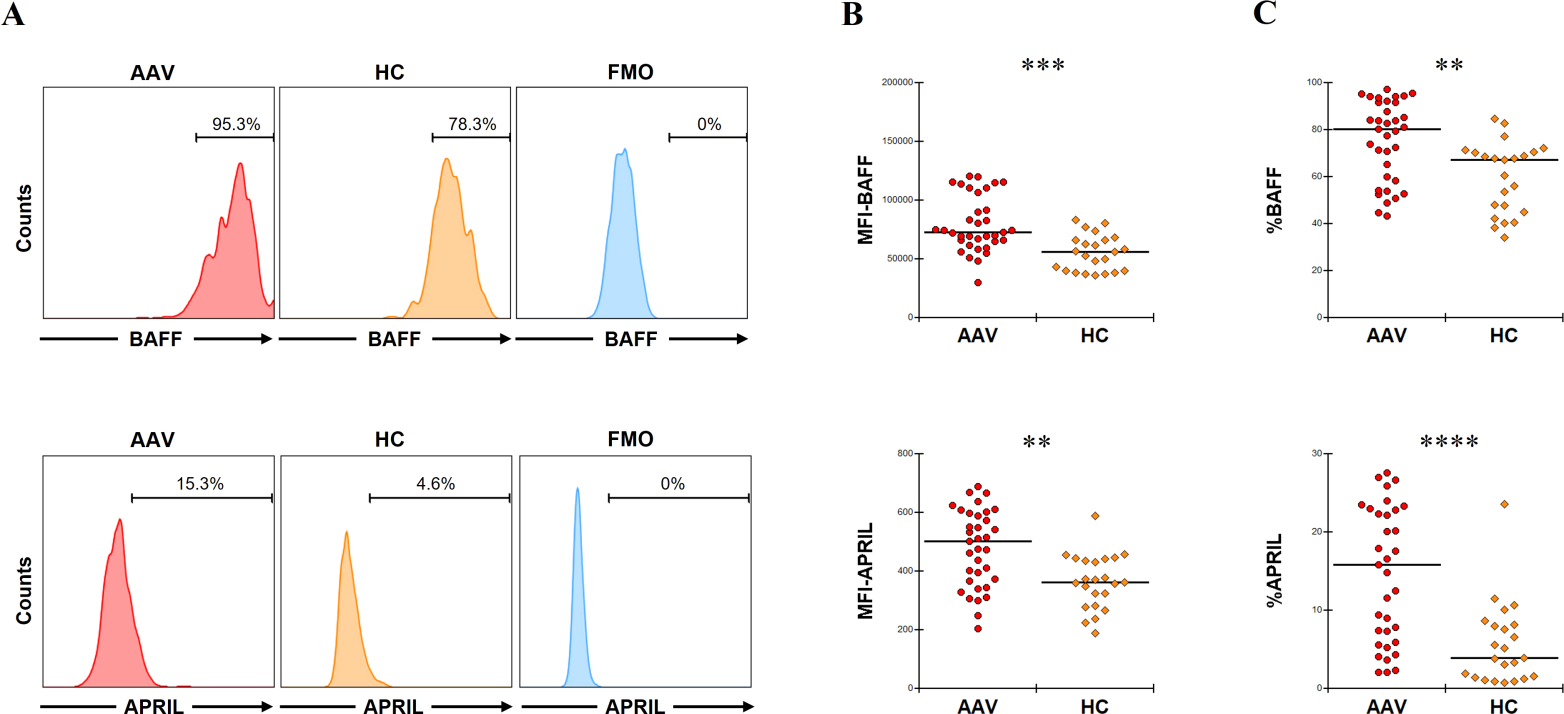
B-cell activation factor of the tumor necrosis factor family (BAFF) and a proliferation-inducing ligand (APRIL) expression in untreated CD14+ cells. **(A)** The representative histograms of BAFF or APRIL expression in CD14+ cells without any treatment. Range bars indicate the proportion of BAFF or APRIL positive in CD14+ cells. **(B)** The median fluorescence intensity (MFI) of BAFF or that of APRIL in CD14+ cells without any treatment between patients with antineutrophil cytoplasmic antibody-associated vasculitis (AAV, n = 35) and healthy controls (HC, n = 22). **(C)** The proportion of BAFF or that of APRIL in CD14+ cells without any treatment between patients with AAV and HC. FMO, fluorescence minus one. **p < 0.005, ***p < 0.001, ****p < 0.0001.

The MFIs of BAFF and APRIL in CD14+ cells were significantly higher in patients with AAV than in HC (*p* = 0.0001 and *p* = 0.001, respectively) (**Figure 1A, B**). The proportions of BAFF and APRIL in CD14+ cells were also significantly higher in patients with AAV than in HC (BAFF: median [IQR], 80.2 [59.2–91.8] % vs. 67.5 [47.9–70.6] %, *p* = 0.001; APRIL: 15.8 [6.6–22.9] % vs. 4.5 [1.7–8.2] %, *p* < 0.0001) (**Figure 1A, C**). In the ROC curve analyses for determining the significant proportions of BAFF and APRIL in patients with AAV (the cutoff proportion) using AAV as the objective variable and HC as the control variable, the AUCs for BAFF and APRIL were 0.764 (sensitivity 0.686, specificity 0.783, *P* < 0.0001) and 0.808 (sensitivity 0.657, specificity 0.827, *P* < 0.0001), respectively (**Supplementary Figure 2**). The cutoff proportions of BAFF and APRIL in CD14+ cells were estimated to 70.9% and 9.0%, respectively. In the analysis for the impacts on clinical findings, the MFI of BAFF in CD14+ cells was positively correlated with BVAS (coefficient 0.0002, 95%CI 0.0001 to 0.0003, *p* = 0.0004), while that of APRIL was inversely correlated with BVAS (coefficient –0.0159, 95%CI –0.0309 to –0.0009, *p* = 0.039) (**Table 1**). Additionally, the MFI of BAFF in CD14+ cells was positively associated with pulmonary and renal involvements (OR 1.0001, 95%CI 1.0000 to 1.0002, *p* = 0.037; OR 1.0001, 95%CI 1.0000 to 1.0001, *p* = 0.015, respectively) (**Supplementary Table 3**). The MFI of APRIL in CD14+ cells was inversely associated with renal involvement (OR 0.9925, 95%CI 0.9851 to 0.9999, *p* = 0.047).

**Table 1:** Regression analysis of the impact of BAFF and APRIL expression in monocytes on BVAS in AAV.

Variables	vs. BVAS					
	Simple regression analysis			Multiple regression analysis		
	Coefficient	95% CI	*P* value	Coefficient	95% CI	*P* value
MFI-BAFF	0.0002	0.0001 to 0.0003	0.0002	0.0002	0.0001 to 0.0003	0.0004
MFI-APRIL	-0.0214	-0.0391 to -0.0037	0.019	-0.0159	-0.0309 to -0.0009	0.039

BVAS, Birmingham Vasculitis Activity Score; AAV, ANCA-associated vasculitis; MFI, median fluorescence intensity; CI, confidence interval.

<0.05 was considered statistically significant.

Taken together, patients with AAV showed significantly higher expression of BAFF and APRIL in CD14+ cells compared to HC. These findings may have implication for pulmonary and renal involvement.

### 3.2 BAFF and APRIL expression in CD14+ cells treated with RVL in AAV

**Figure 2:**
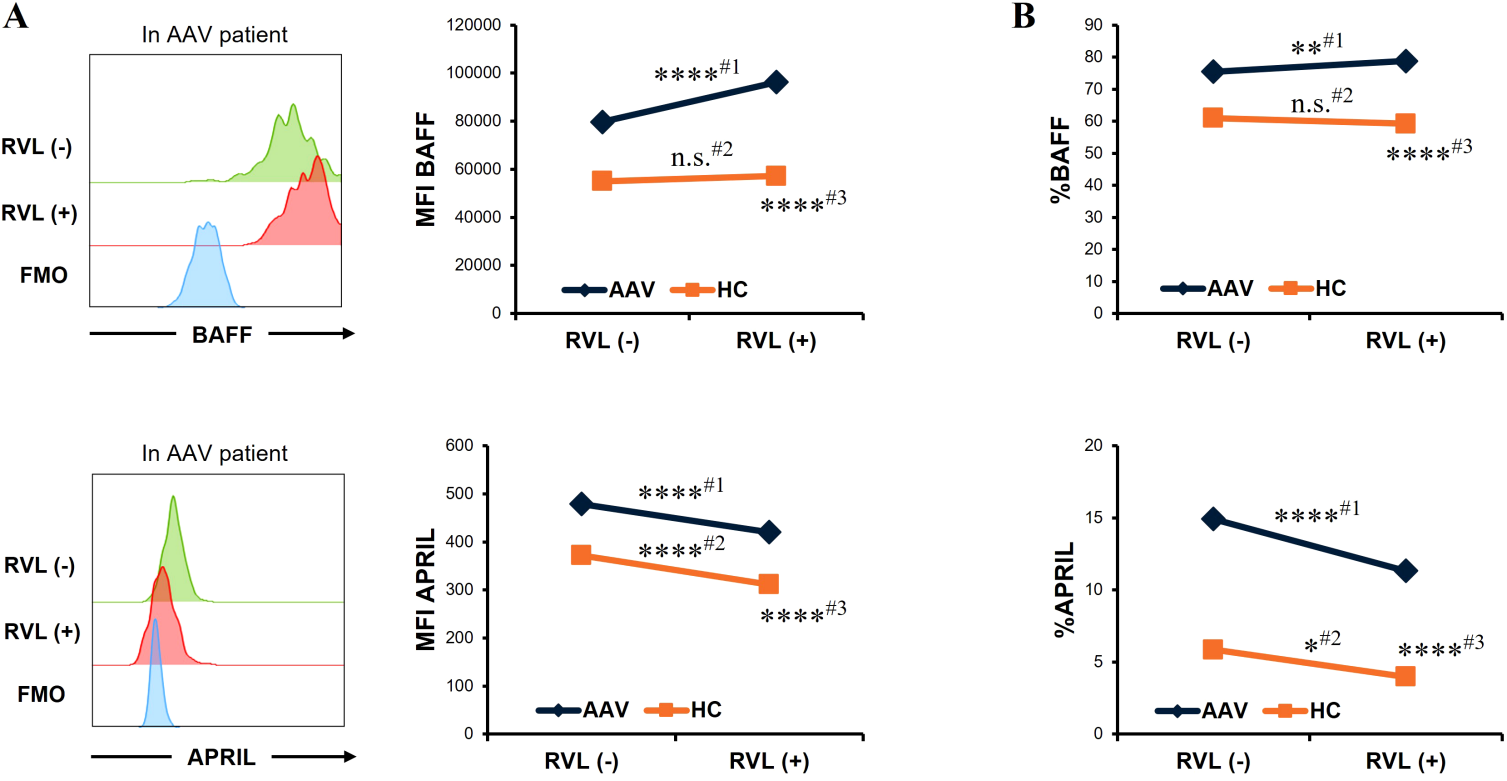
B-cell activation factor of the tumor necrosis factor family (BAFF) and a proliferation-inducing ligand (APRIL) expression in CD14+ cells with and without resveratrol (RVL) treatment. **(A)** Representative histograms of BAFF or APRIL expression in CD14+ cells with and without RVL treatment (left panels) in a patient with antineutrophil cytoplasmic antibody-associated vasculitis (AAV), and median fluorescence intensity (MFI) of BAFF or that of APRIL in CD14+ cells before and after RVL treatment in patients with AAV (n = 35) and in healthy controls (HC; n = 22) (right graphs). **(B)** The proportions of BAFF or APRIL in CD14+ cells before and after RVL treatment in patients with AAV and HC. Comparison before and after RVL treatment in patients with AAV (#1), that in HC (#2), and comparison between patients with AAV and HC after RVL treatment (#3). FMO, fluorescence minus one. *p < 0.05, **p < 0.01, ****p < 0.0001. n.s., not significant.

We evaluated the changes in BAFF and APRIL expression in CD14+ cells after RVL treatment. In patients with AAV, the MFI of BAFF was significantly higher in CD14+ cells treated with RVL than in those not treated with RVL (*p* < 0.0001), whereas that of APRIL was significantly lower in CD14+ cells treated with RVL than in those not treated with RVL (*p* < 0.0001) (**Figure 2A**). The proportion of BAFF was significantly increased in CD14+ cells treated with RVL compared with those without RVL treatment (*p* = 0.007), whereas that of APRIL was significantly decreased in CD14+ cells treated with RVL compared with those without RVL treatment (*p* < 0.0001) (**Figure 2B**). In the analysis of HC, the MFI and the proportion of BAFF were not significantly different between CD14+ cells with and without RVL treatment (*p* = 0.452 and *p* = 0.137, respectively), whereas those of APRIL were significantly lower in CD14+ cells treated with RVL than in those without RVL treatment (*p* < 0.0001 and *p* = 0.026, respectively) (**Figure 2B**). In CD14+ cells treated with RVL, the MFIs of BAFF and APRIL were significantly higher in patients with AAV than in HC (*p* < 0.0001 and *p* < 0.0001, respectively) (**Figure 2A**), and the proportions of BAFF and APRIL were also significantly higher in patients with AAV than in HC (*p* < 0.0001 and *p* < 0.0001, respectively) (**Figure 2B**).

Overall, RVL treatment increased intracellular BAFF expression in patients with AAV, while it reduced intracellular APRIL expression in both patients with AAV and HC.

### 3.3 ROS expression in CD14+ cells treated with RVL and tempol in AAV

**Figure 3:**
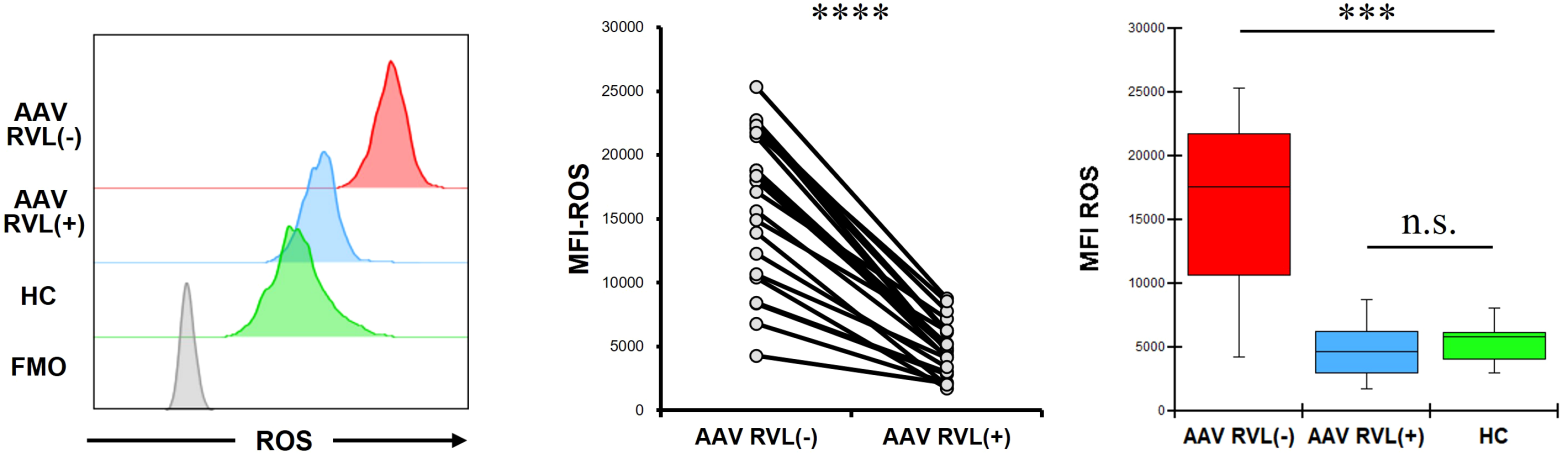
Reactive oxygen species (ROS) expression in CD14+ cells with and without resveratrol (RVL) treatment. The left panel shows representative histograms of ROS in CD14+ cells with or without RVL treatment from a patient with antineutrophil cytoplasmic antibody-associated vasculitis (AAV), and ROS expression in those without RVL treatment from healthy control (HC). The middle graph displays the median fluorescence intensity (MFI) of ROS in CD14+ cells before and after RVL treatment in patients with AAV (n = 22). The right graph indicates the comparisons of the MFIs of ROS in CD14+ cells with and without RVL treatment from patients with AAV (n = 22), and those without RVL treatment from HC (n = 9). FMO, fluorescence minus one. ***p < 0.001, ****p < 0.0001. n.s., not significant.

The MFI of ROS in CD14+ cells without RVL treatment was significantly higher in patients with AAV than in HC (*p* = 0.0002) (**Figure 3**). In patients with AAV, the MFI of ROS was significantly lower in CD14+ cells treated with RVL than in CD14+ cells not treated with RVL (*p* < 0.0001). The MFI of ROS in CD14+ cells treated with RVL from patients with AAV was not significantly different from that in CD14+ cells without RVL treatment from HC (*p* = 0.622).

**Figure 4:**
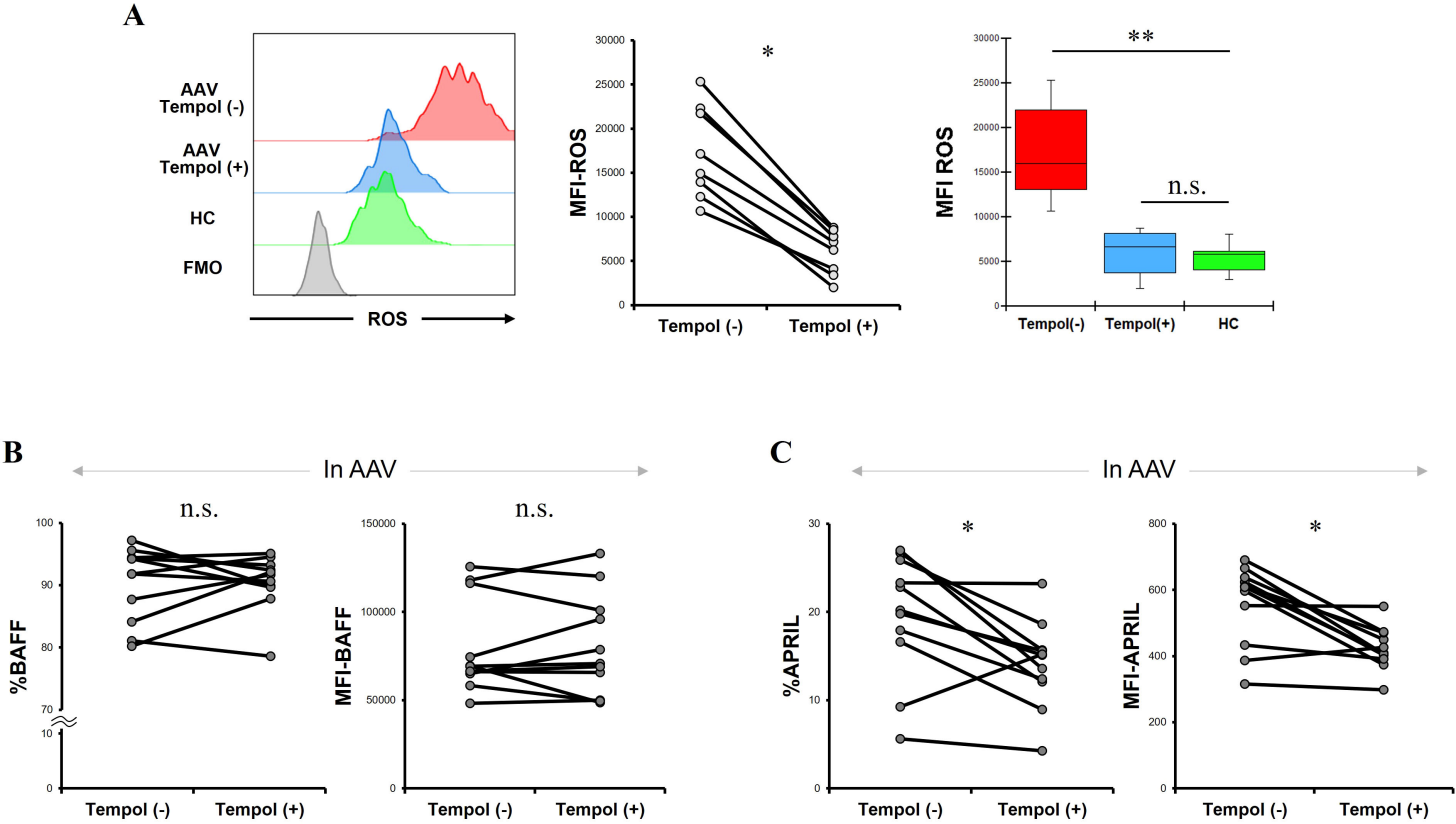
Reactive oxygen species (ROS), B-cell activation factor of the tumor necrosis factor family (BAFF), and a proliferation-inducing ligand (APRIL) expression in CD14+ cells with and without tempol treatment. **(A)** The left panel shows representative histograms of ROS in CD14+ cells with or without tempol treatment from a patient with antineutrophil cytoplasmic antibody-associated vasculitis (AAV), and ROS expression in those without tempol treatment from a healthy control (HC). The middle graph displays the median fluorescence intensity (MFI) of ROS in CD14+ cells before and after tempol treatment in patients with AAV (n = 8). The right graph indicates the comparisons of the MFIs of ROS in CD14+ cells with and without RVL treatment from patients with AAV, and those without RVL treatment from HC (n = 9). **(B)** The proportion and median fluorescence intensity (MFI) of BAFF, and **(C)** those of APRIL in CD14+ cells before and after tempol treatment in patients with AAV. FMO, fluorescence minus one. *p < 0.05, **p < 0.005. n.s., not significant.

Additionally, we treated CD14+ cells with tempol in patients with AAV. The MFI of ROS was significantly lower in CD14+ cells treated with tempol than in those not treated with tempol (*p* = 0.012) (**Figure 4**). The MFI of ROS in CD14+ cells treated with tempol from patients with AAV was not significantly different from that in CD14+ cells not treated with tempol from HC (*p* = 0.721). The proportion and MFI of BAFF in CD14+ cells treated with tempol were not significantly different from those in CD14+ cells not treated with tempol (*p* = 0.929 and *p* = 0.859, respectively). The proportion and MFI of APRIL in CD14+ cells treated with tempol were significantly lower than those in CD14+ cells not treated with tempol (*p* = 0.016 and *p* = 0.008, respectively).

In patients with AAV, treatments with RVL and tempol significantly reduced intracellular ROS levels, while also demonstrating a significant decrease in intracellular APRIL expression.

### 3.4 NF-κB and its relationship with BAFF and APRIL expression in CD14+ cells

**Figure 5:**
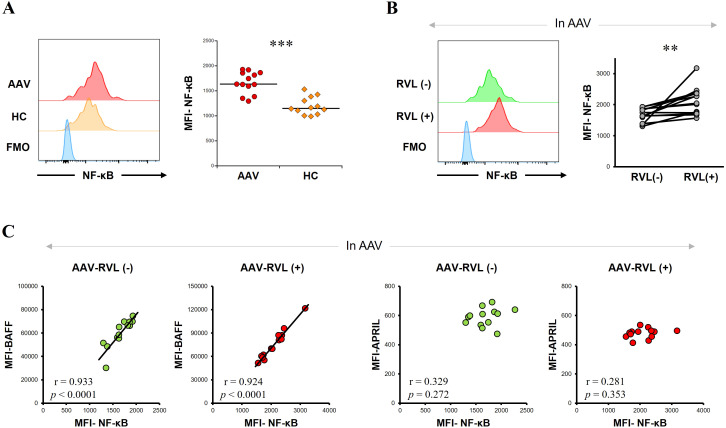
Nuclear factor-κB (NF-κB) expression and its correlations with B-cell activation factor of the tumor necrosis factor family (BAFF) or a proliferation-inducing ligand (APRIL) in CD14+ cells. **(A)** The left panel is representative histograms of NF-κB expression in CD14+ cells without any treatment, and the right graph is the comparison of median fluorescence intensity (MFI) of NF-κB in CD14+ cells without any treatment between patients with antineutrophil cytoplasmic antibody-associated vasculitis (AAV, n = 13) and healthy controls (HC, n = 12). **(B)** The left panel is representative histograms of NF-κB expression in CD14+ cells with and without resveratrol (RVL) treatment, and the right graph shows MFI of NF-κB in CD14+ cells before and after RVL treatment in patients with AAV. **(C)** Correlations between MFI of NF-κB and that of BAFF or that of APRIL in CD14+ cells with and without RVL treatment in patients with AAV. FMO, fluorescence minus one. **p < 0.005, ***p < 0.001.

LPS for stimulating monocytes, namely Toll-like receptor (TLR) 4 ligation stimulation, participates in activating its downstream signaling, including NF-κB pathways. Moreover, the LPS-induced TLR4 binding cascade is involved in BAFF/APRIL signaling through the activation of the NF-κB pathway (31, 32). Accordingly, we investigated NF-κB expression in CD14+ cells from patients with AAV and HC. In untreated CD14+ cells, the MFI of NF-κB was significantly higher in patients with AAV than in HC (*p* = 0.0002) (**Figure 5A**). In patients with AAV, the MFI of NF-κB was significantly higher in CD14+ cells treated with RVL than in those not treated with RVL (*p* = 0.002) (**Figure 5B**). The MFI of NF-κB was significantly correlated with that of BAFF in both populations of CD14+ cells with and without RVL treatment (*p* < 0.0001 and *p* < 0.0001, respectively) but was not correlated with that of APRIL (*p* = 0.272 and *p* = 0.353, respectively) (**Figure 5C**). In the evaluation of HC, the MFI of NF-κB was not significantly different in CD14+ cells treated with RVL and in those without RVL treatment (*p* = 0.136); nevertheless, in both CD14+ cells with and without RVL treatment, the MFI of NF-κB was significantly correlated with that of BAFF (*p* = 0.0001 and *p* = 0.0003, respectively) and with that of APRIL (*p* < 0.0001 and *p* = 0.047, respectively) (**Supplementary Figure 3**).

Taken together, in patients with AAV, intracellular NF-κB expression was significantly higher compared to HC, and this expression significantly increased with RVL treatment. Patients with AAV exhibited significant correlations between intracellular BAFF and NF-κB expression, regardless of RVL treatments; however, no correlations were found between intracellular APRIL and NF-κB expression.

### 3.5 Changes of BAFF and APRIL expression in CD14+ cells after treatment with SN50 in AAV

**Figure 6:**
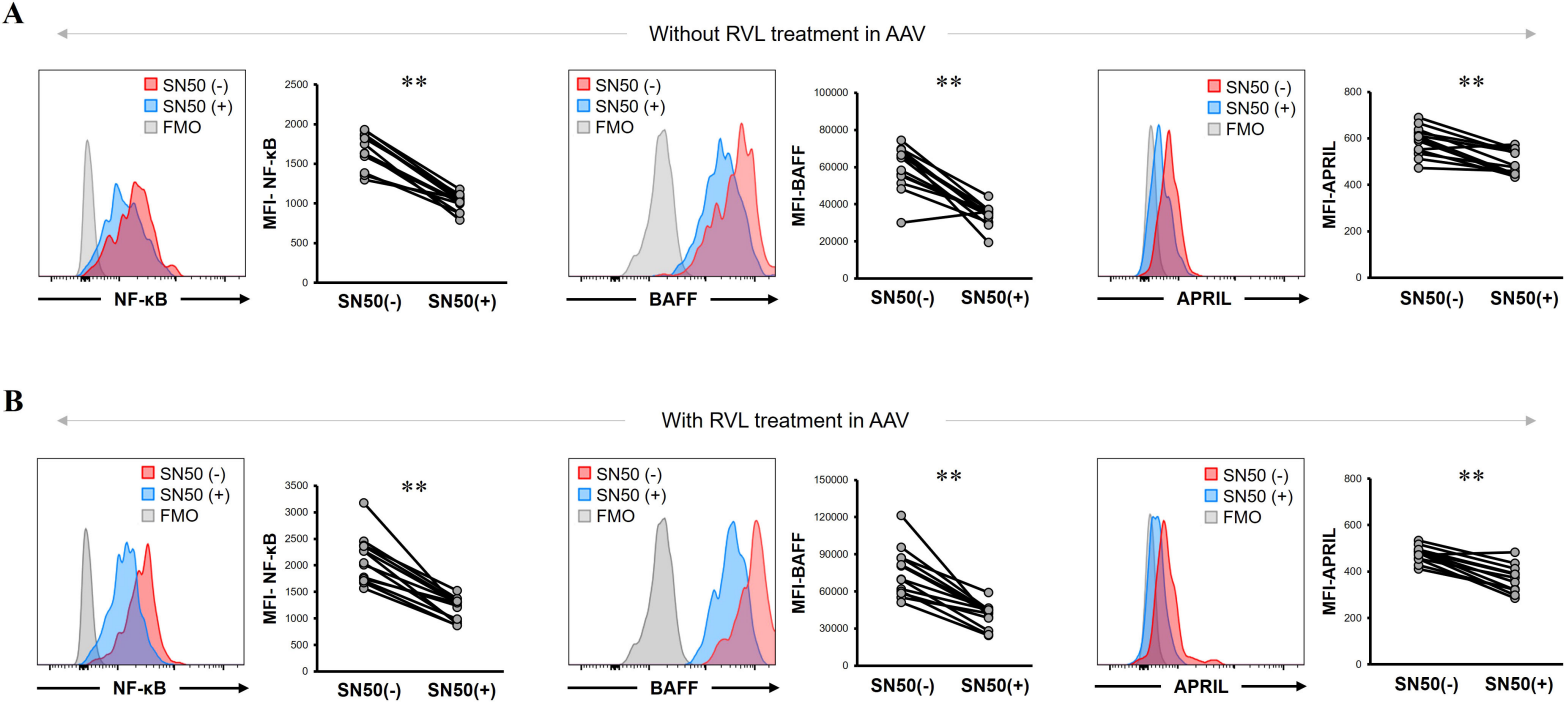
B-cell activation factor of the tumor necrosis factor family (BAFF) and a proliferation-inducing ligand (APRIL) expression in CD14+ cells treated with nuclear factor-κB (NF-κB) inhibitor with and without resveratrol (RVL) treatment in patients with antineutrophil cytoplasmic antibody-associated vasculitis. **(A)** Representative histograms and median fluorescence intensity (MFI) of NF-κB, BAFF, and APRIL in untreated CD14+ cells with and without SN50 treatment. **(B)** Representative histograms and median fluorescence intensity (MFI) of NF-κB, BAFF, and APRIL in CD14+ cells with and without SN50 treatment, which were simultaneously treated with RVL. FMO, fluorescence minus one. **p < 0.005.

**Figure 7:**
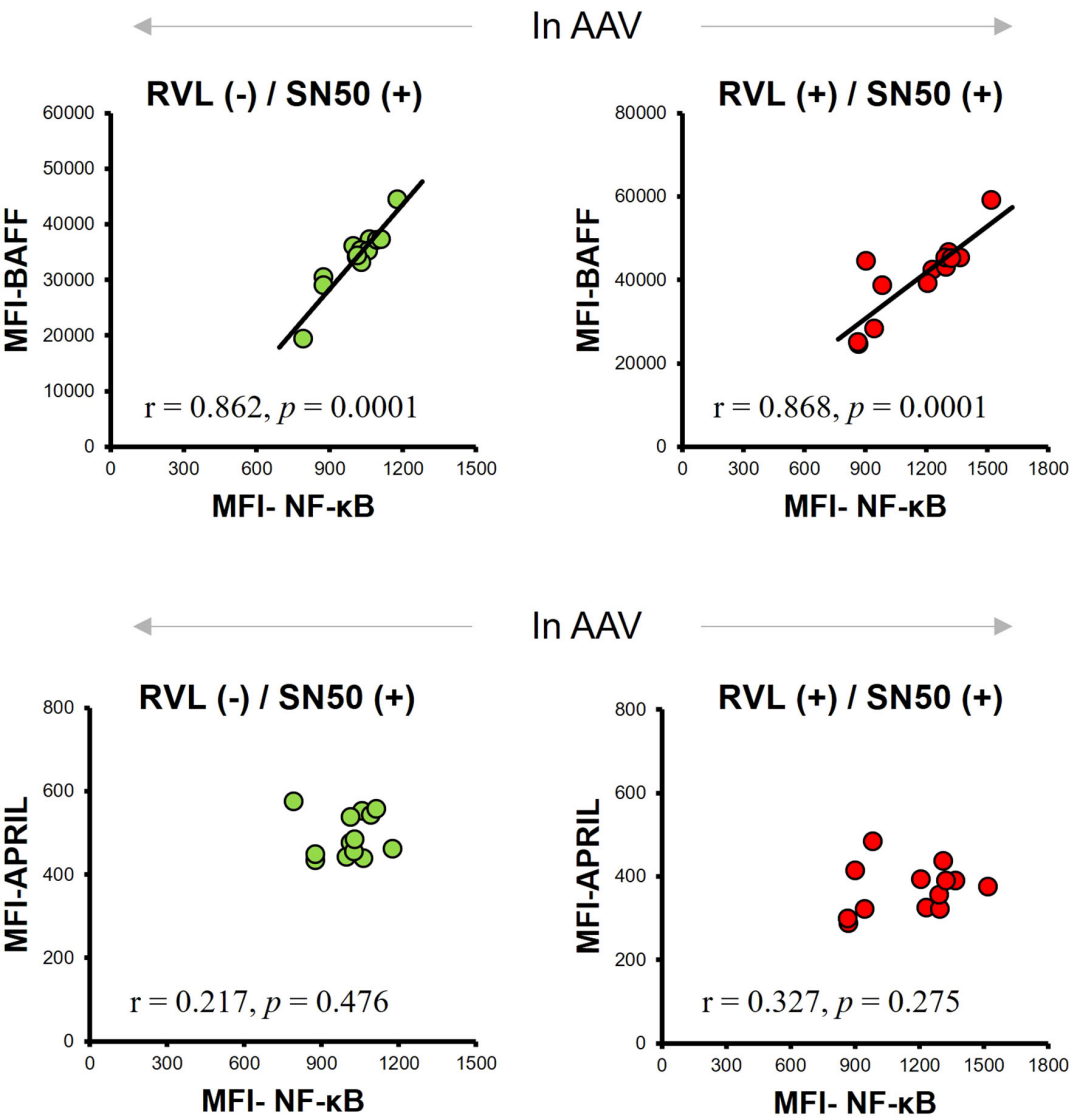
Correlations between median fluorescence intensity (MFI) of nuclear factor-κB (NF-κB), B-cell activation factor of the tumor necrosis factor family (BAFF), and a proliferation-inducing ligand (APRIL) expression in CD14+ cells with and without resveratrol (RVL) treatment, which were simultaneously treated with SN50, in patients with antineutrophil cytoplasmic antibody-associated vasculitis (AAV).

Next, we analyzed the BAFF and APRIL expression in CD14+ cells by inhibiting NF-κB expression using SN50 in patients with AAV. The MFI of NF-κB was significantly lower after SN50 treatment than before SN50 treatment in CD14+ cells without RVL treatment (*p* = 0.0015) (**Figure 6A**) and in those treated with RVL (*p* = 0.0015) (**Figure 6B**). In CD14+ cells without RVL treatment, the MFIs of BAFF and APRIL were significantly lower after SN50 treatment than before SN50 treatment (*p* = 0.0019 and *p* = 0.0024, respectively) (**Figure 6A**). In CD14+ cells treated with RVL, the MFIs of BAFF and APRIL were also significantly lower after SN50 treatment than those before SN50 treatment (*p* = 0.0015 and *p* = 0.0019, respectively) (**Figure 6B**). The MFI of BAFF was positively correlated with that of NF-κB in both CD14+ cells with and without RVL treatment after SN50 treatment (*p* = 0.0001 and *p* = 0.0001, respectively), whereas the MFI of APRIL was not correlated with that of NF-κB (*p* = 0.275 and *p* = 0.476, respectively) (**Figure 7**).

The SN50 treatment significantly reduced the expression of BAFF and APRIL, together with decrease in NF-κB expression, regardless of RVL treatment. Of those, a significant correlation was found between BAFF and NF-κB expression; however, no correlation was observed between APRIL and NF-κB expression.

## 4 Discussion

This study demonstrated significant increases in BAFF and APRIL expression in monocytes from patients with AAV. Additionally, our study suggests that intracellular BAFF production is robustly associated with the NF-κB signal in AAV because significant correlations between NF-κB and BAFF expression were universally found in monocytes with and without inhibition treatment of NF-κB. RVL treatment induced intracellular BAFF production in monocytes and increased NF-κB expression in patients with AAV. However, no significant differences in BAFF expression were observed in monocytes from healthy individuals, regardless of RVL treatment. This demonstrated that the NF-κB signal is pivotal for producing BAFF, and RVL may act on inducing BAFF expression through the NF-κB signal in AAV. The NF-κB binding site is located in the BAFF promoter region, resulting in regulating BAFF expression (33, 34). Some anti-inflammatory reagents, such as the phenolic compound chlorogenic acid and chicoric acid, have been found to suppress intracellular BAFF expression by regulating NF-κB (34, 35), explaining that the NF-κB signal pathway is implicated in BAFF expression. In the experiment for activating pattern recognition receptors using OM-85, which is a standardized lysate of human airways bacteria but does not act on LPS receptors (36), induction of BAFF via the activation of NF-κB was demonstrated in dendritic cells (37). However, the effects of RVL on BAFF and NF-κB in monocytes were not observed in healthy individuals. This suggests that the effect of RVL on the immunity of AAV, which increases intracellular BAFF expression through the stimulation of NF-κB signaling, is different from that of the physiological environment.

Our results also suggested that NF-κB is a physiologically crucial factor for both producing BAFF and APRIL in monocytes because intracellular expression of NF-κB was significantly correlated with that of BAFF and APRIL in healthy individuals. In patients with AAV, exposure to RVL and SN50 conversely affected NF-κB expression, whereas SN50 decreased it. However, both treatments significantly reduced APRIL expression in monocytes. This indicates that other factors may play a more significant role in intracellular APRIL production, although the influence of NF-κB on APRIL production remains in patients with AAV. Ultimately, the redox reactions mediated by RVL and tempol were found to play a critical role in reducing intracellular APRIL expression.

A significant increase in ROS expression was observed in monocytes from patients with AAV compared with those in healthy individuals. AAV pathogenesis is robustly associated with excessive oxidative stress, and activated neutrophils are pivotal for ROS production (38). Persistent increased ROS expression in immunocompetent cells leads to the excessive promotion of immune responses, such as the production of inflammatory cytokines and other inflammatory factors, in autoimmune diseases, including AAV (21, 39). Monocytes play a crucial role as innate immune priming cells in mediating the pathological processes associated with AAV (40). Moreover, ROS production can be induced in monocytes primed by ANCA (41). Intracellular APRIL expression was reduced when monocytes were treated with RVL in both patients with AAV and healthy individuals, whereas the suppressive effect of RVL on BAFF expression was not observed, suggesting that RVL can constitutively regulate APRIL production in monocytes. RVL is known to act as an antioxidant and anti-inflammatory mediator and has multimodal properties, including regulation of oxidative stress, modulation of the energy metabolic system and nutrient sensing, and activation of senescence-related genetic and epigenetic factors (42, 43). In the immune system, RVL plays a role in the intracellular regulation of the immune pathway by suppressing not only oxidative stress but also various key signaling molecules. To our knowledge, the impact of ROS on intracellular APRIL expression has not been reported, although a previous study has indicated that oxidative stress induces BAFF expression in adipocytes (44). However, our study demonstrated that intracellular ROS expression was not associated with BAFF production in monocytes from patients with AAV. In contrast, treatment with tempol, a representative ROS-scavenging nitroxide compound that acts as a superoxide dismutase (45), led to a significant decrease in APRIL expression and reduced ROS levels. This suggests that increased intracellular ROS expression may significantly influence APRIL production in monocytes, as two different types of ROS-scavengers, RVL and tempol, yielded similar results.

Our study indicated a significant correlation between BAFF expression in monocytes and BVAS, along with renal and pulmonary involvements, suggesting that BAFF-expressing monocytes may be biological marker of affecting disease activity in AAV. A previous study has shown that BVAS was significantly correlated with serum levels of BAFF, although it was not correlated with APRIL levels in patients with ANCA-associated renal vasculitis (8). In contrast, no significant correlations between serum BAFF or APRIL levels and BVAS have been reported in other AAV studies (11, 12). To the best of our knowledge, a specific AAV manifestation significantly associated with serum BAFF or APRIL levels has not yet been demonstrated. The increased production of BAFF and APRIL can contribute to the development of AAV by promoting autoreactive B cells, whereas BAFF and APRIL are produced by various types of myeloid cells, including monocytes, macrophages, neutrophils, and dendritic cells (5, 6, 46). Some studies have reported that the dominant infiltration of monocytes/macrophages in biopsied renal tissues in ANCA-associated glomerulonephritis (40, 47, 48). Given our results and the associated pathological aspects, investigating the implication of BAFF-expressing monocytes in the targeted organ, including pulmonary and renal lesions, may clarify a more precise immunopathologic mechanism of AAV. APRIL expression in monocytes was found to be inversely associated with BVAS and renal involvement. We assumed that APRIL expression in monocytes might be inversely regulated, depending on the predominant production of APRIL in other types of immunocompetent cells and the disease activity.

Our study had some limitations. First, we focused exclusively on the expression of intracellular BAFF and APRIL in CD14+ monocytes, although other immunocompetent cells are also involved in producing BAFF and APRIL. Second, we conducted *in vitro* experiments using LPS stimulation on PBMCs to investigate the expression of BAFF, APRIL, and other relevant factors in monocytes. It may be necessary to consider multiple priming factors in the immune systems to fully understand the signal transduction pathways that lead to BAFF and APRIL production. Third, our experiment used PBMCs for treatments with various reagents, indicating that monocytes may be influenced by other types of immune cells. To better understand the specific intracellular mechanisms involved in BAFF/APRIL signaling, an alternative experimental design that isolates and treats monocytes alone may be necessary. Fourth, the very small number of samples from patients with AAV and healthy individuals might be insufficient to analyze the relationships between intracellular BAFF/APRIL expression and clinical findings.

In conclusion, significantly increased expression of BAFF and APRIL in monocytes was observed in patients with AAV, and these were positively and inversely correlated with disease activity, respectively. RVL led to increased BAFF expression and decreased APRIL expression in monocytes from patients with AAV. Moreover, NF-κB was involved in the correlative impact on increasing BAFF expression in monocytes in patients with AAV, and RVL exerted its effect by promoting NF-κB expression. In contrast, a redox reaction induced by RVL led to a decrease in APRIL expression in monocytes from patients with AAV. Our results indicated intracellular features of BAFF/APRIL signaling in the monocytes of patients with AAV. It is necessary to clarify the precise intracellular signaling pathways that produce BAFF/APRIL in all relevant immunocompetent cells that contribute to the development of AAV.

## Data Availability

The raw data supporting the conclusions of this article will be made available by the authors, without undue reservation.
