# Supplementary material for: Impact of NF-κB and reactive oxygen species on intracellular BAFF/APRIL expression in ANCA-associated vasculitis: focusing on the effect of resveratrol
Source: Front Immunol. 2025 Jun 4;16:1586158. doi: 10.3389/fimmu.2025.1586158 (PMC12174086; doi:10.3389/fimmu.2025.1586158)
Supplement: Supplementary file 1 [file SupplementaryFile1.pptx]

## Slide 1
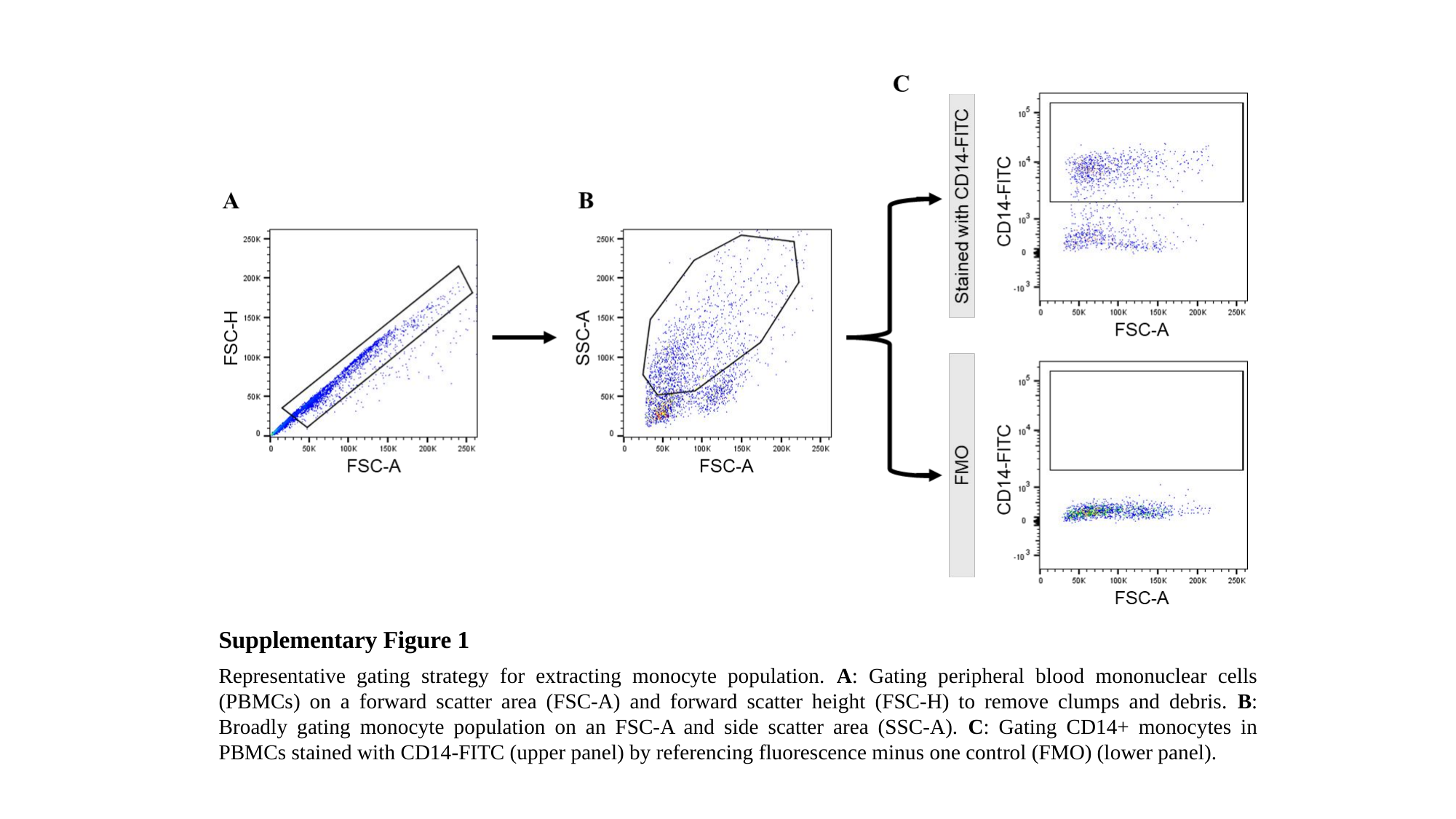

Supplementary Figure 1
Representative gating strategy for extracting monocyte population. A: Gating peripheral blood mononuclear cells (PBMCs) on a forward scatter area (FSC-A) and forward scatter height (FSC-H) to remove clumps and debris. B: Broadly gating monocyte population on an FSC-A and side scatter area (SSC-A). C: Gating CD14+ monocytes in PBMCs stained with CD14-FITC (upper panel) by referencing fluorescence minus one control (FMO) (lower panel).

## Slide 2
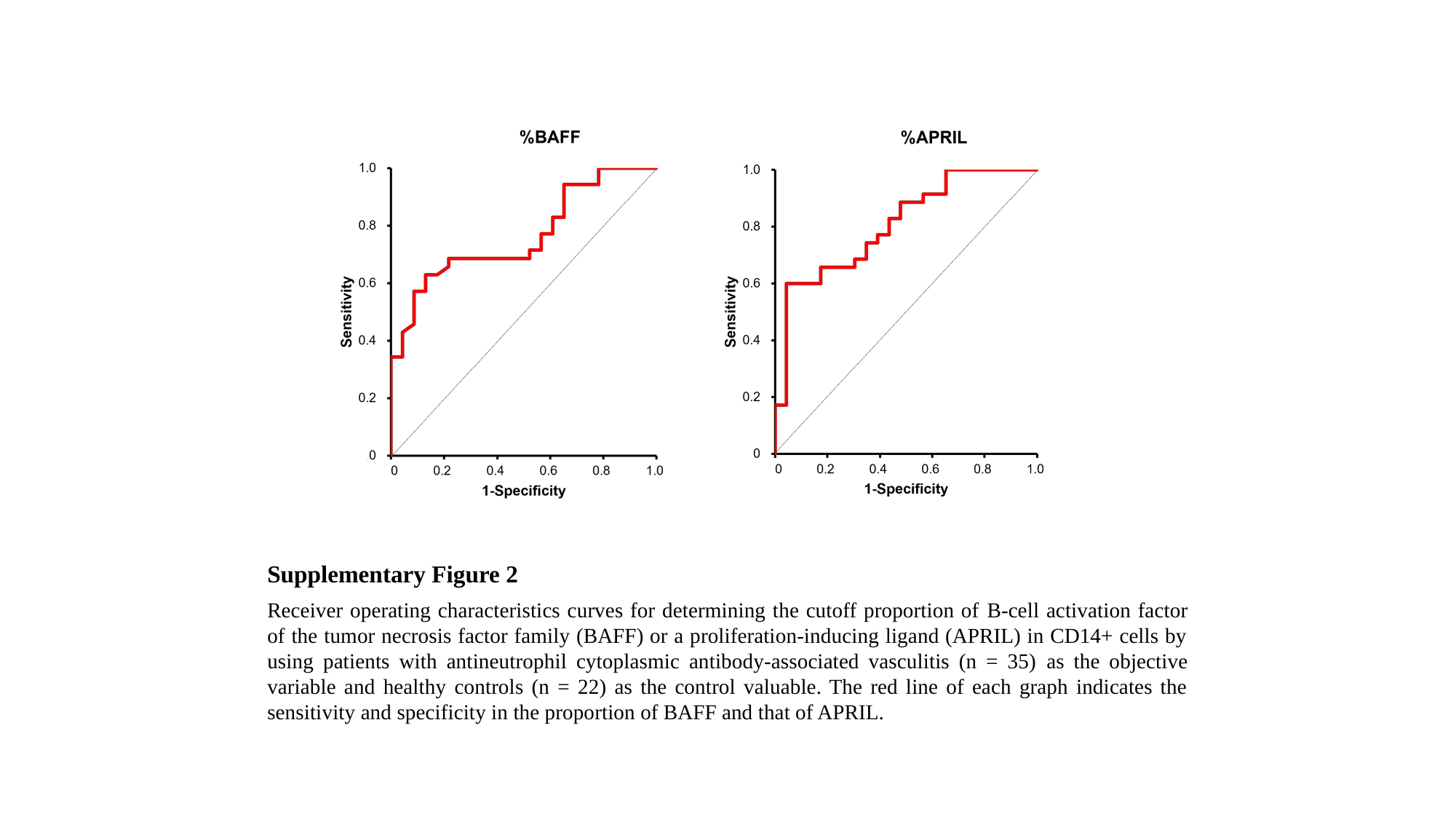

Supplementary Figure 2
Receiver operating characteristics curves for determining the cutoff proportion of B-cell activation factor of the tumor necrosis factor family (BAFF) or a proliferation-inducing ligand (APRIL) in CD14+ cells by using patients with antineutrophil cytoplasmic antibody-associated vasculitis (n = 35) as the objective variable and healthy controls (n = 22) as the control valuable. The red line of each graph indicates the sensitivity and specificity in the proportion of BAFF and that of APRIL.

## Slide 3
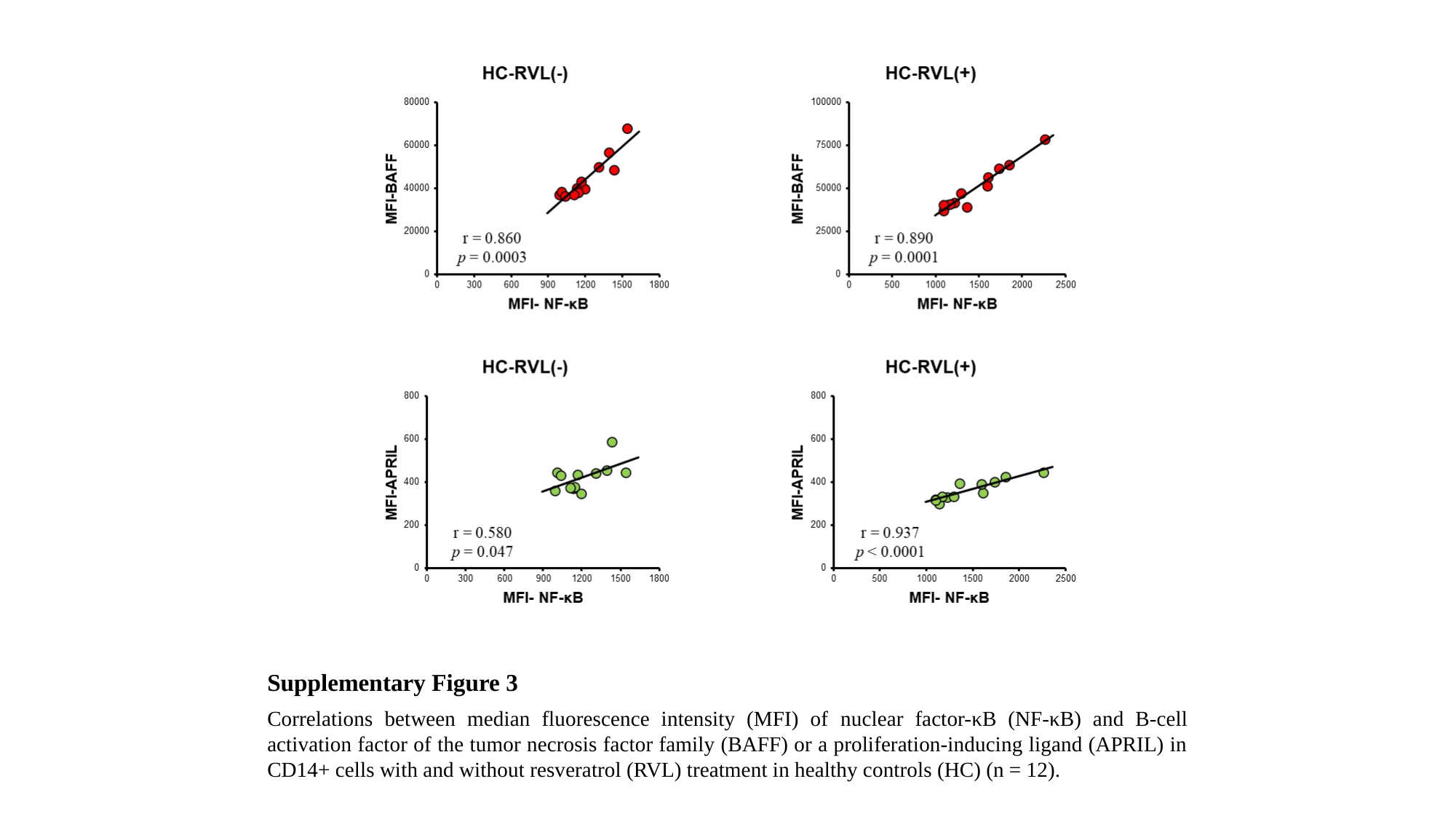

Supplementary Figure 3
Correlations between median fluorescence intensity (MFI) of nuclear factor-κB (NF-κB) and B-cell activation factor of the tumor necrosis factor family (BAFF) or a proliferation-inducing ligand (APRIL) in CD14+ cells with and without resveratrol (RVL) treatment in healthy controls (HC) (n = 12).
