# Supplementary material for: Impact of NF-κB and reactive oxygen species on intracellular BAFF/APRIL expression in ANCA-associated vasculitis: focusing on the effect of resveratrol
Source: Front Immunol. 2025 Jun 4;16:1586158. doi: 10.3389/fimmu.2025.1586158 (PMC12174086; doi:10.3389/fimmu.2025.1586158)
Supplement: Supplementary file 2 [file SupplementaryFile2.docx]

**Supplementary Table 1** Epidemiological and clinical findings

|  |  | **AAV** | **HC** | ***p*-value** |
| --- | --- | --- | --- | --- |
|  | Number | 35 | 22 |  |
|  | Median (IQR), year | 71 (66‒75) | 64 (51‒72) | 0.085 |
|  | Female (%) | 22 (63) | 12 (55) | 0.587 |
| **Classification**, n (%) | |  |  |  |
|  | MPA | 21 (60) |  |  |
|  | GPA | 14 (40) |  |  |
| **Symptoms**, n (%) | |  |  |  |
|  | Fever (more than 38°C) | 21 (60) |  |  |
|  | Arthritis/arthralgia | 10 (29) |  |  |
|  | Myalgia | 11 (31) |  |  |
|  | Weight loss (more than 2 kg) | 11 (31) |  |  |
|  | Cutaneous | 5 (14) |  |  |
|  | Mucous membranes/eyes | 7 (20) |  |  |
|  | ENT | 15 (43) |  |  |
|  | Pulmonary | 25 (71) |  |  |
|  | Cardiovascular | 2 (6) |  |  |
|  | Abdominal | 1 (3) |  |  |
|  | Renal | 21 (60) |  |  |
|  | Nervous system | 14 (40) |  |  |
| **BVAS**, median (IQR) | | 15 (9‒19) |  |  |
| **Laboratory data** | |  |  |  |
|  | MPO-ANCA, n (%) | 29 (83) |  |  |
|  | PR3-ANCA, n (%) | 5 (14) |  |  |
|  | White blood cells, median (IQR), /µL | 11480 (8875‒14225) |  |  |
|  | Neutrophils, median (IQR), /µL | 9430 (7238‒12264) |  |  |
|  | Lymphocytes, median (IQR), /µL | 1100 (865‒1712) |  |  |
|  | C-reactive protein, median (IQR), mg/dL | 9.61 (4.2‒13.4) |  |  |
|  | eGFR, median (IQR) | 0.72 (0.58‒0.84) |  |  |

AAV, ANCA-associated vasculitis; HC, healthy controls; IQR, interquartile range; ENT, ear, nose, and throat; BAS, the Birmingham Vasculitis Activity Score; MPO, Myeloperoxidase; PR3, Proteinase 3; eGFR, estimated glomerular filtration rate.

**Supplementary Table 2** Antibody clones for flow cytometry

| **Antibody** | **Clone** |
| --- | --- |
| FITC-conjugated anti-CD14 | M5E2 |
| PE-conjugated anti-BAFF | 1D6 |
| APC-conjugated anti-APRIL | REA347 |
| PE/Cy7-conjugated anti-NF-κB | K10-895.12.50 |

**Supplementary Table 3**

Regression analysis of the impacts of BAFF and APRIL on relevant manifestations in AAV

| Outcome variable | |  | **Univariate logistic regression** | | |  | **Multivariate logistic regression** | | |
| --- | --- | --- | --- | --- | --- | --- | --- | --- | --- |
|  |  |  | OR | 95% CI | *p* value |  | OR | 95% CI | *p* value |
| Fever (more than 38°C) | | | |  |  |  |  |  |  |
|  | MFI-BAFF |  | 1.00001 | 0.99998 to 1.00005 | 0.317 |  | 1.00002 | 0.99998 to 1.00005 | 0.317 |
|  | MFI-APRIL |  | 0.99976 | 0.99455 to 1.00499 | 0.928 |  | 1.00032 | 0.99491 to 1.00575 | 0.907 |
| Arthritis/arthralgia | | | |  |  |  |  |  |  |
|  | MFI-BAFF |  | 1.00001 | 0.99998 to 1.00004 | 0.559 |  | ― | ― | ― |
|  | MFI-APRIL |  | 1.00929 | 1.00125 to 1.01741 | 0.023 |  | ― | ― | ― |
| Myalgia | |  |  |  |  |  |  |  |  |
|  | MFI-BAFF |  | 1.00003 | 0.99999 to 1.00006 | 0.067 |  | ― | ― | ― |
|  | MFI-APRIL |  | 1.00178 | 0.99579 to 1.00780 | 0.560 |  | ― | ― | ― |
| Weight loss (more than 2 kg) | | | |  |  |  |  |  |  |
|  | MFI-BAFF |  | 1.00003 | 0.99999 to 1.00006 | 0.069 |  | ― | ― | ― |
|  | MFI-APRIL |  | 1.00173 | 0.99576 to 1.00775 | 0.569 |  | ― | ― | ― |
| Cutaneous | |  |  |  |  |  |  |  |  |
|  | MFI-BAFF |  | 1.00001 | 0.99997 to 1.00005 | 0.499 |  | ― | ― | ― |
|  | MFI-APRIL |  | 1.00766 | 0.99797 to 1.01744 | 0.121 |  | ― | ― | ― |
| Mucous membranes/eyes | | | |  |  |  |  |  |  |
|  | MFI-BAFF |  | 0.99999 | 0.99996 to 1.00003 | 0.782 |  | ― | ― | ― |
|  | MFI-APRIL |  | 1.00007 | 0.99355 to 1.00665 | 0.981 |  | ― | ― | ― |
| Ear, nose, and throat | | | |  |  |  |  |  |  |
|  | MFI-BAFF |  | 1.00003 | 0.99999 to 1.00006 | 0.071 |  | ― | ― | ― |
|  | MFI-APRIL |  | 0.99535 | 0.98982 to 1.00091 | 0.101 |  | ― | ― | ― |
| Pulmonary | |  |  |  |  |  |  |  |  |
|  | MFI-BAFF |  | 1.00011 | 1.00002 to 1.00019 | 0.020 |  | 1.0001 | 1.0000 to 1.0002 | 0.037 |
|  | MFI-APRIL |  | 0.99406 | 0.98739 to 1.00077 | 0.083 |  | 0.9963 | 0.9889 to 1.0038 | 0.333 |
| Cardiovascular | |  |  |  |  |  |  |  |  |
|  | MFI-BAFF |  | 1.00013 | 0.99989 to 1.00036 | 0.281 |  | ― | ― | ― |
|  | MFI-APRIL |  | 1.00165 | 0.98899 to 1.01448 | 0.799 |  | ― | ― | ― |
| Abdominal | |  |  |  |  |  |  |  |  |
|  | MFI-BAFF |  | ― | ― | ― |  | ― | ― | ― |
|  | MFI-APRIL |  | ― | ― | ― |  | ― | ― | ― |
| Renal | |  |  |  |  |  |  |  |  |
|  | MFI-BAFF |  | 1.00007 | 1.00001 to 1.00013 | 0.011 |  | 1.0001 | 1.0000 to 1.0001 | 0.015 |
|  | MFI-APRIL |  | 0.99197 | 0.98534 to 0.99864 | 0.018 |  | 0.9925 | 0.9851 to 0.9999 | 0.047 |
| Nervous system | |  |  |  |  |  |  |  |  |
|  | MFI-BAFF |  | 0.99999 | 0.99996 to 1.00002 | 0.596 |  | ― | ― | ― |
|  | MFI-APRIL |  | 0.99766 | 0.99224 to 1.00311 | 0.399 |  | ― | ― | ― |

AAV, ANCA-associated vasculitis; OR, odds ratio; CI, confidence interval; MFI, median fluorescence intensity.
